# A Novel Method for Mouse Retinal Temperature Determination Based on ERG Photoresponses

**DOI:** 10.1007/s10439-017-1872-y

**Published:** 2017-06-15

**Authors:** Marja Pitkänen, Ossi Kaikkonen, Ari Koskelainen

**Affiliations:** Department of Neuroscience and Biomedical Engineering, Aalto University School of Science, P.O. Box 12200, 00076 Aalto, Finland

**Keywords:** Electroretinography, Retina, Retinal pigment epithelium, Kinetics, Temperature-dependence

## Abstract

This study introduces a novel retinal temperature determination method based on the temperature dependent properties of photoresponses recorded by electroretinography (ERG). The kinetics and amplitudes of ERG photoresponses depend on retinal temperature. Additionally, raising retinal temperature increases the probability of long-wavelength photon absorption, which manifests as temperature dependence of photoreceptor sensitivity. In this study we extract a number of features that represent these properties from the a- and b-waves of mouse *ex vivo* ERG flash responses and construct three multivariable regression models between temperature and the selected features. The performance of these models was evaluated against a separate test dataset and for two of the models, an RMS temperature determination error of less than 0.50 °C could be reached. Our results demonstrate that the method can be successfully used for reliable retinal temperature determination *ex vivo*. The method, reflecting the temperature of distal retina, can be applied also in the estimation of retinal pigment epithelium temperature.

## Introduction

Heating the retinal pigment epithelium (RPE) offers a potential treatment for several retinal diseases, such as choroidal melanoma, age-related macular degeneration, diabetic macular edema, and central serous chorioretinopathy.^16^,^18^,^20^,^27^,^28^,^35^ However, the lack of an effective method for measuring the temperature of the retina and the RPE constitutes one of the main obstacles in the development of heating treatment procedures. Overheating photoreceptors and RPE leads to cell damage and cell death, whereas underheating vitiates the therapeutic effect. Furthermore, testing appropriate heating routines in a repetitive manner demands adequate control of temperature.^16^

The most straightforward method for RPE heating applies irradiation with wavelengths mainly absorbed by the melanin pigment in the RPE and negligibly by the tissues of the frontal eye. The temperature rise is thus strongest in the RPE, whereas behind it, on the distal side, the temperature drops steeply due to strong choroidal circulation.^22^ On the proximal side of the RPE, the temperature drop is expected to be more gradual due to the less efficient and more distant retinal circulation.^36^ The outer segments (OSs) of photoreceptors lie on the proximal side of the RPE, partially embedded in it. Therefore, the temperature of the OSs closely coincides with that of the RPE, enabling the temperature estimation of the RPE and the distal retina based on photoresponses originating in the OSs. The method demonstrated in this paper primarily employs the temperature dependent changes that arise in the OS layer or in the subsequent bipolar cell layer.

This study proposes a novel method for retinal/RPE temperature determination by electroretinography (ERG). ERG is a common assessment tool in basic and clinical retinal research and it can be recorded both *ex vivo* from an isolated retina or *in vivo* from the surface of the cornea.^6^ The method is based on temperature dependent properties of ERG photoresponses: kinetics, absolute sensitivity, and relative sensitivity at long wavelengths. Photoresponse kinetics becomes faster as the temperature rises (see Refs. 1,12,25 for single cell recordings and Refs. 4,21,34 for ERG) due to the acceleration of both the activation and deactivation processes of photoresponses. Secondly, the absolute sensitivity, defined as the amplitude of a photoresponse per stimulus strength unit, is inversely proportional to temperature near mammalian body temperature.^1^,^4^,^12^,^21^,^25^ Additionally, at long wavelengths, where the photon energy alone is insufficient to excite the visual pigment, thermal energy can supplement the excitation and increase the probability of photon absorption.^11^,^31^,^32^ This phenomenon manifests as an increase in photoreceptor sensitivity with temperature for long-wavelength stimuli in comparison to shorter-wavelength stimuli, and we call it long-wavelength relative sensitivity, LRS, in this paper.

 We applied scotopic *ex vivo* ERG recordings from isolated mouse retinas to investigate the feasibility and to estimate the accuracy of the proposed temperature determination method. With this configuration, the temperature of the retina could be changed rapidly and controlled precisely, enabling accurate determination of the temperature dependencies of ERG photoresponse features. The data presented here serves as reference for the calibration of this temperature determination model for *in vivo* mouse ERG and, above all, provides a proof of concept for successful retinal temperature determination based on ERG photoresponses.

In recent years, RPE heating treatment studies have increasingly focused on methods with moderately low increase in temperature (e.g. Refs. 13,29). The therapeutic mechanisms remain unclear, however, it has been proposed that the thermal stress in the RPE is one of the factors responsible for the beneficial treatment outcomes.^30^ These treatments aim at staying below the damage threshold of neural retina in order to preserve vision, which emphasizes the need for precise control over the temperature. Thus, the temperature determination method proposed in this paper would be suitable and beneficial for such treatments. In addition to temperature estimation, the ERG signal provides an indicator of the condition of neural retina during the heating treatment. Other possible applications for the proposed method include temperature-controlled retinal drug delivery from thermosensitive polymers.

## Materials and Methods

### ERG Photoresponses

Retinal photoresponses originate in the outer segments of rods and cones, where the information of photon absorption is transformed into an electrical signal through a G-protein cascade known as phototransduction (see e.g. Ref. 23). As a result of photon absorption, the membrane current of the outer segment diminishes causing the inside of the cell to hyperpolarize. ERG records changes in the ohmic voltage associated with the radial currents in the retinal extracellular space. ERG photoresponse is a combination of superimposed components arising from the electrical activity of different retinal cell types. The leading edge of a response to a bright flash of light, the a-wave, mainly reflects the changes in the inflowing current of rod and/or cone outer segments.^7^,^24^ The subsequent component, the b-wave, originates from the activity of ON-bipolar cells.^33^ Overlapping with these apparent waveforms, the slow PIII component arising in the Müller glial cells contributes to the signal.^2^ The temperature determination method described in this paper is based on the analysis of mouse rod a- and b-wave amplitudes and kinetics.

### Ethical Approval

The use and handling of the animals were in accordance with the Finnish Act on Animal Experimentation 2006 and guidelines of the Animal Experiment Board in Finland.

### *Ex vivo* ERG Experiments

**Figure 1:**
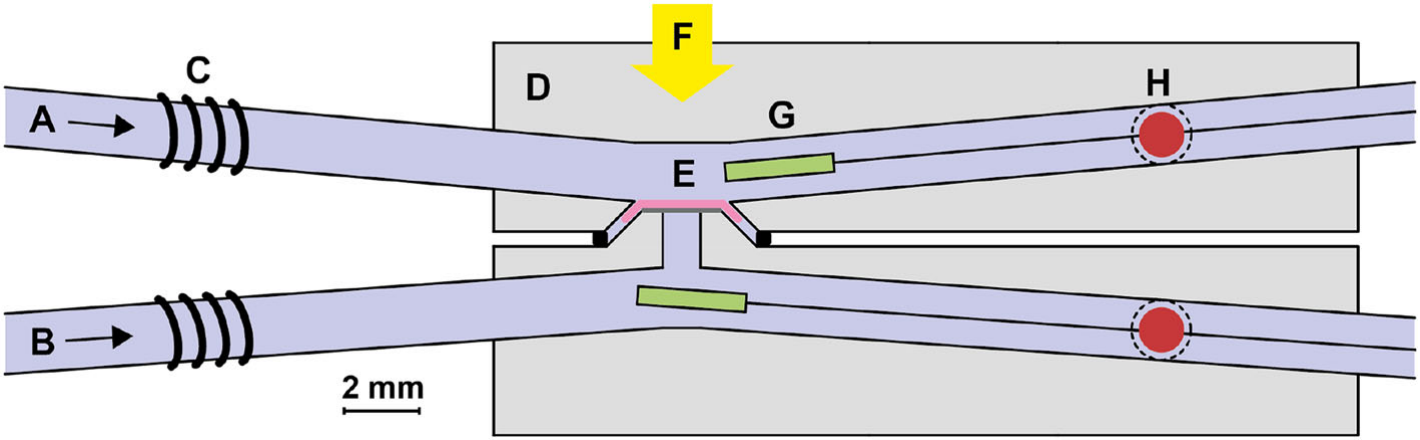
Recording setup. (a) Upper solution channel; (b) lower solution channel; (c) resistive heating wire; (d) specimen holder (polycarbonate); (e) retina on a filter paper; (f) light stimulation; (g) thermistor; (h) recording electrode (inside a solution channel perpendicular to the image plane).

Two to four months old male and female mice of strain C57BL/6J were dark-adapted overnight. The animals were sacrificed by CO_2_ inhalation and cervical dislocation, the eyes were enucleated and bisected along the equator. The sclera and the RPE were removed in cooled Ringer’s solution under dim red light and the isolated retina was placed photoreceptors upwards in a specimen holder (modified from Ref. 4) illustrated in Fig. 1. The specimen holder was optimized for fast temperature changes e.g. by minimizing its volume. A constant flow (ca. 4 ml min^−1^) of Ringer’s solution perfused the retina on both sides: on top of photoreceptors and under the thin filter paper on which the retina was placed. Ringer’s solution consisted of (mM): Na^+^, 115.6; K^+^, 3.3; Mg^2+^, 2.0; Ca^2+^, 1.0; Cl^−^, 124.9; glucose, 10.0; EDTA, 0.01; HEPES, 10.0. 4.8 mM NaOH and 20 mM NaHCO_3_ was added to the solution and the pH was balanced to 7.5 by bubbling with carbogen (95% O_2_, 5% CO_2_). Leibovitz culture medium L-15, 0.72 mg ml^−1^, was introduced to improve the viability of the retina in all experiments. A small amount of BaCl_2_ (5–10 *µ*M), which inhibits the potassium currents of Müller cells, was added to the solution in some experiments where the slow PIII component greatly suppressed the b-wave amplitudes of dim flash responses.^21^ All chemicals were purchased from Sigma-Aldrich.

### Temperature Control

The temperature of the retina was monitored with a small thermistor (Tewa Termico TT5-10KC3-72, diameter ~0.5 mm) placed inside the upper solution channel so that the distance between the thermistor tip and the recording area was less than 2 mm. The retinal temperature was controlled by changing the temperature of the upper solution flow. Resistive heating wires were coiled around the solution tubes and the heating power was controlled with a custom-made PID-controller. The temperature of the lower solution was held constant at 37 °C and monitored using a similar thermistor. Our goal was to mimic the temperature distribution of the retina during heating treatments where the heating light is mainly absorbed into the melanin pigments of RPE and conducted from there to the distal direction.

### Recording and Light Stimulation

The transretinal potential was recorded with two Ag/AgCl pellet electrodes (World Precision Instruments EP2) directly connected to upper and lower perfusion solutions. The signal was low-pass filtered (Bessel 8-pole with *f*_c_ = 500 Hz) and sampled at 10 kHz with a voltage resolution of 0.25 *μ*V. Light stimuli with homogeneous illumination to the distal side of the retina were provided by a dual-beam optical system (adapted from Ref. 4). Light flashes of 2 ms were generated with a shutter (Oriel 76992, Newport) for both laser paths, the midpoint of the flash indicating the zero time for the recordings. A 532 nm laser diode module (Power Technology IQ5C(532-100)L74, ~130 mW) and a 780 nm laser diode (Roithner Lasertechnik RLT780-150GS, ~150 mW) served as light sources. The uniformity of the beam was confirmed with a camera-based beam profiler (Spiricon Laser Beam Diagnostics SP503U). Stimulus strengths were controlled by attenuating the light with calibrated neutral density filters and wedges. The absolute intensity of the unattenuated laser light (photons mm^−2^ s^−1^) incident on the retina was measured with a calibrated photodiode (EG&G HUV-1000B; calibrated by the National Standards Laboratory of Finland), enabling the determination of the number of isomerizations (R*) produced by the stimulating flash in individual rods as described in Ref. 5.

### Experiment Protocol

The stimulus strengths of bright and dim flashes were assigned for each retina in the beginning of the experiment at 37 °C. Dim flash strengths for both 532 and 780 nm light were selected to induce equal b-waves corresponding to 10–15% of the maximum b-wave amplitude. In this range the photoresponses are linear, i.e. the amplitudes scale linearly with stimulus strength and the response kinetics are unchanged. Bright flash stimuli were generated by the 532 nm light source and the flash strength was selected in the range of 130–210 R* rod^−1^ so that the response had clear a- and b-waves. Stimulus strengths were fine-tuned during experiments if the sensitivity of the photoreceptors changed and the criteria above were no longer fulfilled. During the experiments, retinal temperature was varied in the range 35.0–44.0 °C. At each temperature, the retina was allowed to stabilize before recordings. The waiting times after temperature changes of <1, 1–5, and >5 °C were 90, 120, and 150 s, respectively. Thereafter, eight photoresponses to dim flash stimuli, four per light source, were recorded with 6–7 s interval, followed by a single bright flash response. For each retina, recordings were conducted on average at 15 different temperatures. In *ex vivo* ERG, retinal signaling typically stays stable for several hours enabling long-lasting experiments. However, some deceleration of photoresponse kinetics and variation in photoreceptor sensitivity may take place over time, and the b-wave usually decreases in amplitude. To compensate for these changes, reference photoresponses at 37.0 °C were recorded every ~20 min. Experiments were discontinued if the dim flash b-wave amplitude decreased below 15 *µ*V at 37.0 °C or below 10 *µ*V at any other temperature.

### Pre-processing and Feature Extraction

The dataset of 13 retinas was split into training (*n* = 8 retinas) and test (*n* = 5 retinas) sets randomly. Only the training data set was used in the model development. Four training responses to similar (532 or 780 nm) dim flashes were averaged at each temperature. In the test data set, two versions were created differing in the number of dim flash responses averaged (4 or 1). Bright flash responses were used without averaging both in training and in test data set. The baselines of all responses were first corrected according to the signal offset level preceding the stimulus (0.5 or 1.0 s). Subsequently, several features describing the kinetics and amplitudes were extracted from the responses using Matlab.

Bright flash responses were low-pass filtered (FIR *n* = 50, *f*_c_ = 100 Hz) prior to feature extraction. The a-wave time-to-peak and amplitude were defined as time point $t_{{{\text{a-peak}}}}$ and response value $V(t_{{{\text{a-peak}}}} )$ where the subtraction of response values *V*(*t* + 1 ms) − *V*(*t* − 1 ms) changed sign. Thereafter, the time-to-*X*% of a-wave peak could be defined as the time point $t_{{{\text{a-}}X}}$ at which the comparison $V\left( t \right) <\ X\% \cdot V\left( {t_{{{\text{a-peak}}}} } \right)$ became true. The inflection point was determined as the local minimum of the derivative of a cubic polynomial fitted to the leading edge of the a-wave.

For dim flash response analysis, the time-to-peak *t*_b–peak_ and the amplitude *V*(*t*_b–peak_) were defined based on a quadratic polynomial fit around the b-wave maximum $\left[ {t_{ \hbox{max} } - 15\,{\text{ms}}, \;t_{ \hbox{max} } + 15\,{\text{ms}}} \right]$. The time-to-*X*% features of the b-wave leading edge (*t*_bl–*X*_) and trailing edge (*t*_bl–*X*_) were determined from low-pass filtered responses (FIR-filter, *n* = 600, *f*_c_ = 20 Hz for leading edge features and *n* = 400, *f*_c_ = 30 Hz for trailing edge features) as time points where the comparisons $V\left( t \right) > X\% \cdot V\left( {t_{{{\text{b-peak}}}} } \right), \; t > 0$ and $V\left( t \right) < X\% \cdot V\left( {t_{{{\text{b-peak}}}} } \right), \;t > t_{{{\text{b-peak}}}}$ became true, respectively. The integration time was determined by calculating the integral of filtered (FIR-filter, *n* = 400, *f*_c_ = 30 Hz) response absolute value |*V*(*t*)| by Matlab *trapz*-function from $t = 0$ until the time point where the response reached *V*(*t*) = 0 after the peak. Thereafter, the integral was divided by the amplitude *V*(*t*_b–peak_). Finally, the LRS-feature was computed as a relation of 532 and 780 nm flash response amplitudes $V\left( {t_{{{\text{b-peak}}}} 532} \right)/V\left( {t_{{{\text{b-peak}}}} 780} \right)$. As most of the dim flash response features were not dependent on stimulus wavelength, the final feature values were averaged by combining the corresponding feature values of 532 and 780 nm responses. This averaging was not applied to the b-wave amplitude feature, for which the temperature dependence is unequal for the two stimulus wavelengths. The b-wave amplitude was determined based on the 532 nm response only, due to its steeper temperature dependence. The averaging was not applied for the LRS-feature either, because the feature itself is based on the sensitivity difference at the two wavelengths.

All features were used as relative values to exclude the differences of absolute values between individual retinas. For the training set, this was done by dividing every feature value *x* by the corresponding reference feature value *x*_37_ at 37.0 °C which was determined by linear interpolation in time between the subsequent *x*_37_ values. For the test set, we applied two different normalization procedures. The first one was performed by interpolation as described above. In the second normalization procedure, every feature value was divided only by the most recent reference *x*_37_, in order to mimic a retinal heating treatment session, where information on the future *x*_37_ values is not available.

### Model Development and Validation

Multivariable linear regression models were created by least squares fitting of the equation

1 $$c = X\beta + \varepsilon ,$$

where *c* is the vector of temperatures, *X* is the matrix of feature values (of training set), *β* consists of regression coefficients and *ε* of error terms. A set of possible models was created incrementally by, at each stage, adding the feature which, together with the previously selected features, gave the lowest RMS error for temperature determination in leave-one-out cross-validation with the training dataset. For each model, the Bayesian information criterion (BIC) was calculated using the equation

2 $${\text{BIC}} = n \cdot \ln \left( {\frac{\text{SSE}}{n}} \right) + k \cdot \ln \left( n \right) ,$$

where *n* is the number of observations, SSE is the sum of squared errors, and *k* is the number of estimated parameters in the model. The model with the lowest BIC was selected as the final model, the accuracy of which was evaluated by calculating the errors of temperature determination with a new set of photoresponses (test dataset).

## Results

### Mouse Scotopic Photoresponses

**Figure 2:**
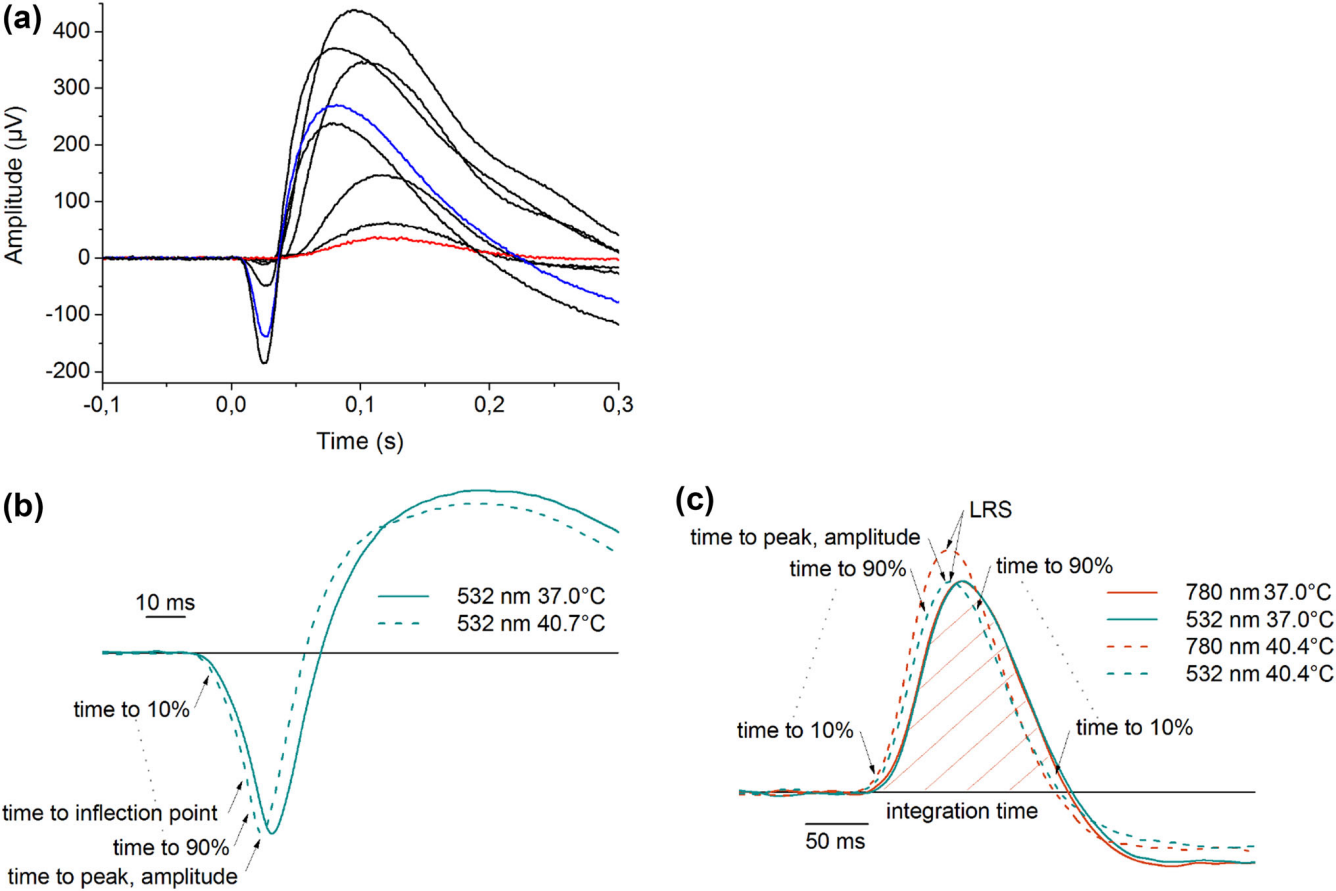
ERG flash responses and feature extraction. (a) Response family of eight dark-adapted flash responses from a representative retina. Flash strengths: 0.17, 0.63, 2.0, 4.0, 9.9, 40, 130, and 200 R* rod^−1^. The red trace and the blue trace represent the dim and bright flash responses chosen for feature extraction, respectively. Each trace represents a single response and no filtering has been performed after the recording; (b) bright flash responses. The response recorded at the higher temperature (40.7 °C, dashed trace) shows accelerated kinetics compared to the one recorded at 37.0 °C with the same stimulus strength (solid trace). The features extracted from bright flash responses representing the kinetics and amplitude of the a-wave are illustrated in the figure. The responses are single recordings that have been normalized to their a-wave peak amplitudes and filtered (FIR, *n* = 50, *f*_c_ = 100); (c) dim flash responses. The responses recorded at higher temperature (40.4 °C, dashed trace) show accelerated kinetics. Furthermore, the response to 780 nm flash stimulus at elevated temperature has higher b-wave amplitude due to increase in photoreceptor sensitivity. From dim flash responses, several features representing the kinetics of b-wave leading and trailing edge, amplitude, integration time and long-wavelength relative sensitivity (LRS) were extracted. The responses are single recordings that have been filtered (FIR, *n* = 400, *f*_c_ = 30 Hz) and normalized according to the b-wave amplitude of the 532 nm flash response separately at both temperatures to elucidate the change in relative sensitivity.

Figure 2a shows a set of eight dark-adapted photoresponses recorded at 37.0 °C. Flash stimuli with increasing strength bring about an emerging b-wave and progressively reveal the a-wave. The red trace represents a dim flash response (~10% of b-wave maximum amplitude) and the blue trace a bright flash response. The temperature dependence of ERG photoresponse kinetics is illustrated in Figs. 2b and 2c. The responses recorded at higher temperatures (dashed traces) show faster onset and earlier recovery when compared to the responses recorded at 37.0 °C (traces) using the same stimulus strengths. The temperature dependence of long-wavelength relative sensitivity is introduced in Fig. 2c. At 37.0 °C, two dim stimulus strengths, one at 780 nm and the other at 532 nm, are adjusted to produce responses with equal b-wave amplitudes. When the retinal temperature is elevated, response amplitudes to the same stimuli become unequal, the response to the 780 nm stimulus having larger amplitude than the response to the 532 nm stimulus. However, the absolute sensitivity of photoreceptors at both wavelengths diminishes when temperature rises (not shown).

### Feature Extraction and Temperature Dependencies

**Figure 3:**
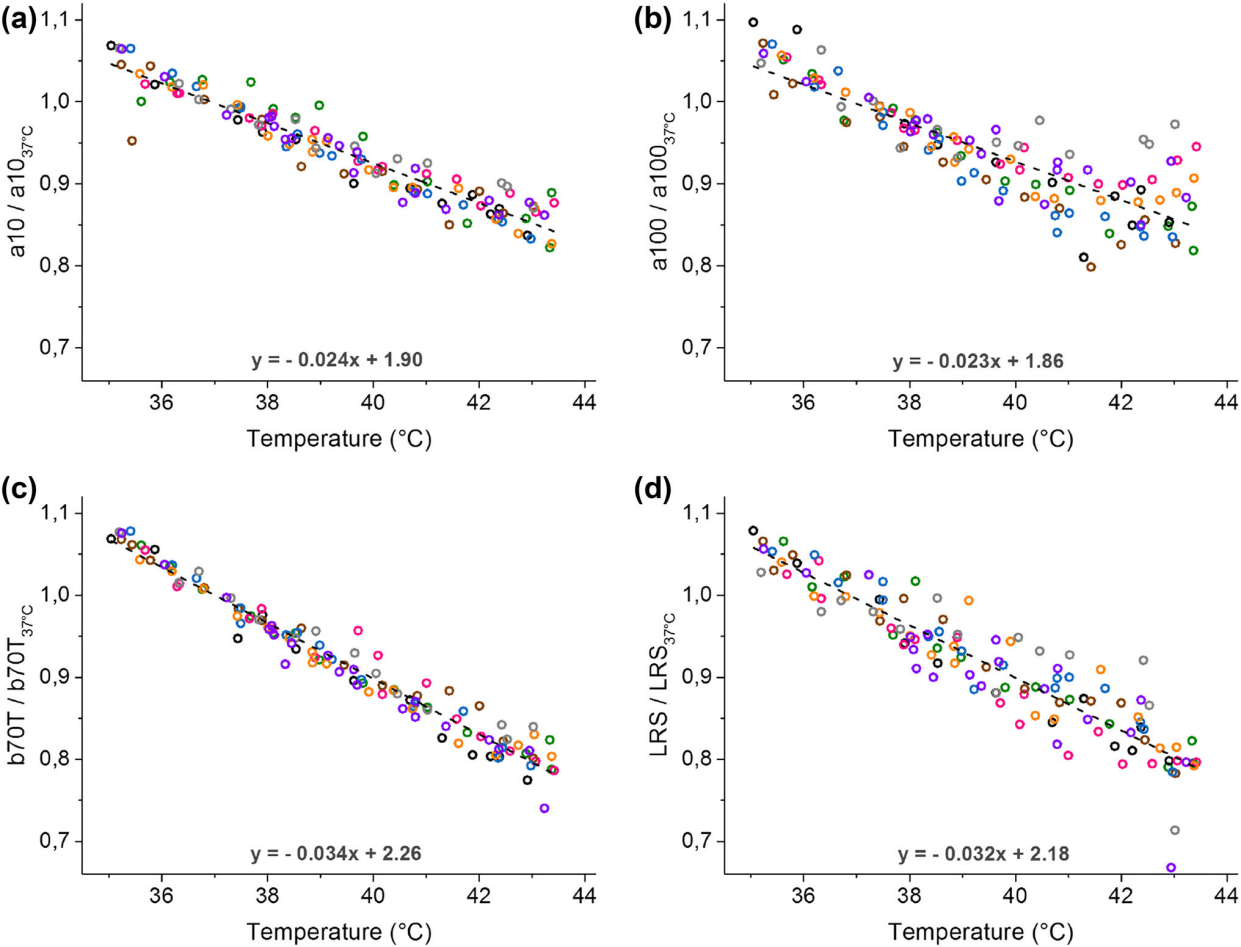
Temperature-dependencies of example features. Each color represents a single retina (*n* = 8 retinas, training dataset) and the dashed line illustrates a least squares fit to the data (slope and intercept of the fit given above the *x*-axis). Feature values have been normalized with corresponding feature values recorded at 37 °C as described in “Materials and Methods”. (a) Time-to-10% of bright flash response a-wave; (b) time-to-peak of bright flash response a-wave; (c) time-to-70% of the peak of dim flash response trailing edge; (d) long-wavelength relative sensitivity.

All 34 features extracted from the photoresponses are illustrated in Figs. 2b and 2c and listed in Table 1. The table also presents the slopes of linear fittings to the training data (as illustrated in Figs. 3a–3d) and root-mean-square deviations between the fit and the data, enabling a crude comparison of the features.

**Table 1:** List of 34 features extracted from bright and dim flash responses.

#^a^	Feature	Response type	Abbr.	Slope^b^	RMSD^b^	RMSD/|Slope|^c^
1	Time to 10% of the peak	Bright flash, a-wave	a10	−0.024	0.019	0.79
2	Time to 20% of the peak	Bright flash, a-wave	a20	−0.025	0.019	0.76
3	Time to 30% of the peak	Bright flash, a-wave	a30	−0.025	0.021	0.84
4	Time to 40% of the peak	Bright flash, a-wave	a40	−0.024	0.024	1.00
5	Time to 50% of the peak	Bright flash, a-wave	a50	−0.023	0.028	1.19
6	Time to 60% of the peak	Bright flash, a-wave	a60	−0.023	0.030	1.32
7	Time to 70% of the peak	Bright flash, a-wave	a70	−0.023	0.032	1.40
8	Time to 80% of the peak	Bright flash, a-wave	a80	−0.023	0.031	1.39
9	Time to 90% of the peak	Bright flash, a-wave	a90	−0.022	0.033	1.47
10	Time to peak	Bright flash, a-wave	a100	−0.023	0.034	1.46
11	Time to inflection point	Bright flash, a-wave	aip	−0.024	0.029	1.22
12	Amplitude	Bright flash, a-wave	aa	−0.040	0.056	1.40
13	Time to 10% of the peak	Dim flash, b-wave leading edge	b10L	−0.024	0.028	1.16
14	Time to 20% of the peak	Dim flash, b-wave leading edge	b20L	−0.024	0.029	1.21
15	Time to 30% of the peak	Dim flash, b-wave leading edge	b30L	−0.024	0.026	1.09
16	Time to 40% of the peak	Dim flash, b-wave leading edge	b40L	−0.024	0.024	1.01
17	Time to 50% of the peak	Dim flash, b-wave leading edge	b50L	−0.024	0.023	0.95
18	Time to 60% of the peak	Dim flash, b-wave leading edge	b60L	−0.025	0.022	0.89
19	Time to 70% of the peak	Dim flash, b-wave leading edge	b70L	−0.025	0.021	0.85
20	Time to 80% of the peak	Dim flash, b-wave leading edge	b80L	−0.025	0.020	0.81
21	Time to 90% of the peak	Dim flash, b-wave leading edge	b90L	−0.026	0.019	0.74
22	Time to peak	Dim flash, b-wave	b100	−0.035	0.022	0.64
23	Time to 90% of the peak	Dim flash, b-wave trailing edge	b90T	−0.033	0.017	0.52
24	Time to 80% of the peak	Dim flash, b-wave trailing edge	b80T	−0.034	0.016	0.47
25	Time to 70% of the peak	Dim flash, b-wave trailing edge	b70T	−0.034	0.016	0.47
26	Time to 60% of the peak	Dim flash, b-wave trailing edge	b60T	−0.034	0.017	0.49
27	Time to 50% of the peak	Dim flash, b-wave trailing edge	b50T	−0.035	0.018	0.51
28	Time to 40% of the peak	Dim flash, b-wave trailing edge	b40T	−0.035	0.019	0.54
29	Time to 30% of the peak	Dim flash, b-wave trailing edge	b30T	−0.035	0.021	0.59
30	Time to 20% of the peak	Dim flash, b-wave trailing edge	b20T	−0.036	0.026	0.73
31	Time to 10% of the peak	Dim flash, b-wave trailing edge	b10T	−0.040	0.050	1.24
32	Integration time	Dim flash, b-wave	bit	−0.044	0.038	0.88
33	Amplitude	Dim flash, b-wave	ba	−0.063	0.085	1.34
34	Long-wavelength relat. sensit.	Dim flash, b-wave	LRS	−0.032	0.032	0.99

^a^Features 1–12 are determined from a single bright flash response, 13–32 and 34 are based on four 532 nm and four 780 nm dim flash responses, and 33 is based on four 532 nm dim flash responses

^b^Slopes of linear fittings to the training data (as illustrated in Figs. 3a–3d) and root mean square deviations (residuals) between the fit and the data. RMSD has the same unit, relative feature value, as the *y*-axes in Fig. 3. Correspondingly, the unit of the slope is relative feature value per degree of Celsius

^c^Low dispersion of feature values around the linear fitting (i.e. small residuals) and a high temperature dependence are beneficial properties for a feature. RMSD/|Slope| reflects the relationship of these properties, low value being desirable

Figure 3 shows the temperature dependencies of four example features giving an overview of the behavior of different types of features. The temperature dependencies appeared generally linear in the temperature range used. Only the a-wave time-to-peak, presented in Fig. 3b, behaved slightly nonlinearly. Linear fittings to the data show about 2–4% change in the feature values per 1 °C (compared to the value at 37 °C).

### Model Development and Validation for All Responses

**Figure 4:**
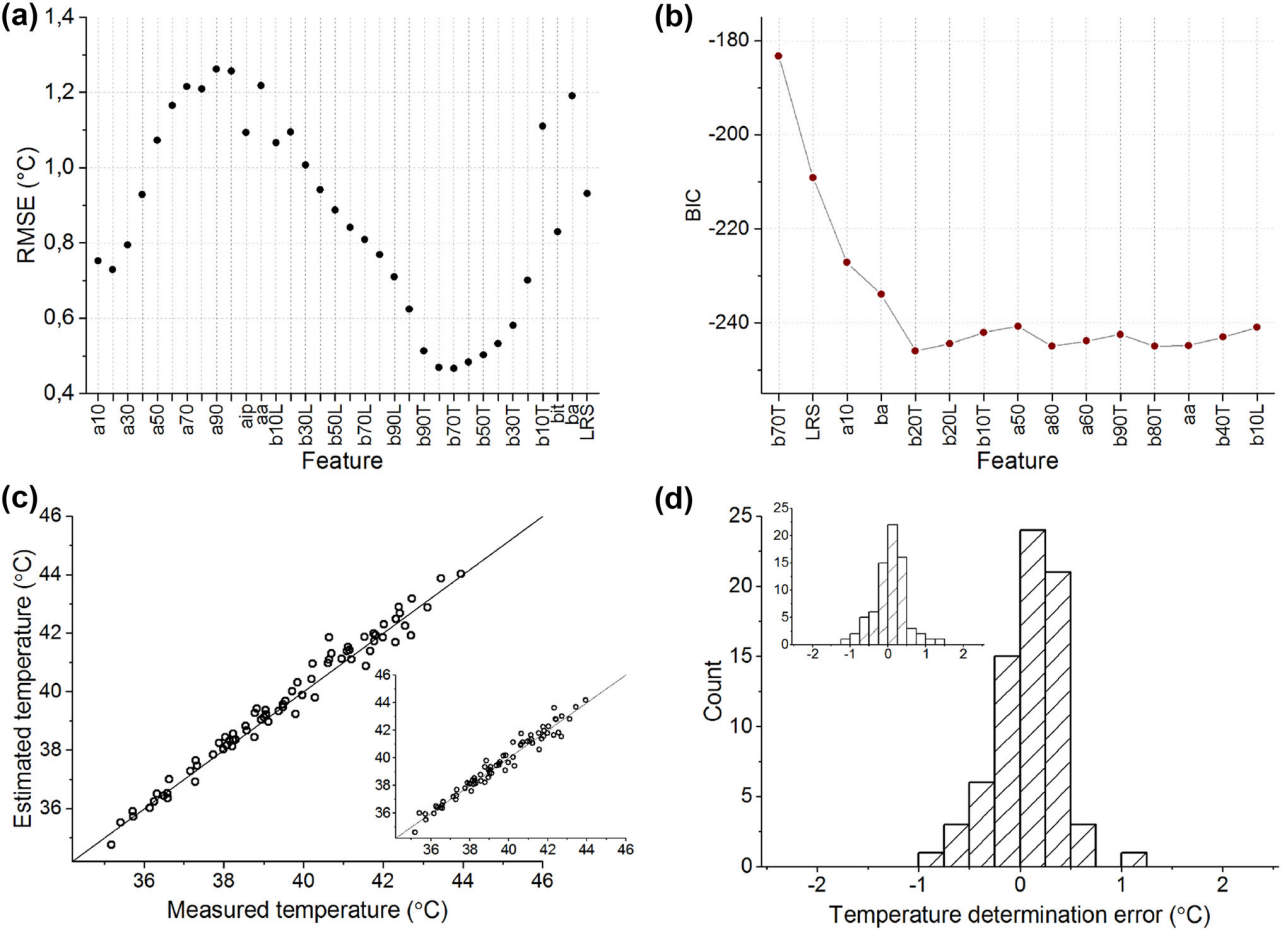
Model development and validation for features extracted from both dim and bright flash responses. (a) Temperature determination errors averaged over cross validations and expressed as RMS values for each linear regression model constructed of a single feature. This figure corresponds to the first step of feature selection: the feature giving the lowest error (b70T) is the first feature included in the final model; (b) behavior of BIC value as features are incrementally added to the final model; (c) relationship between estimated and measured temperatures, when using the constructed model on new data (test set of 5 retinas, 74 data points); (d) histogram of temperature determination errors (*T*_estimated_ − *T*_measured_). In (c) and (d) main figures the feature values of the test set were normalized by interpolated reference values at 37.0 °C. In inset figures the test set feature values were normalized by dividing with the most recent reference feature value.

In order to compare the features, Fig. 4a shows the RMS-error of temperature determination for regression models consisting of each single feature. Errors have been calculated as an average over cross validations. In line with the comparison presented in the last column of Table 1, the features determined from dim flash response trailing edge show highest temperature estimation accuracy.

The temperature determination model was constructed by selecting features to the regression model one-by-one as described in “Materials and Methods” and calculating the BIC value at each step, presented in Fig. 4b. Based on this plot, a model with five features was selected for further analysis. The model consisted of the following features: time-to-70% of b-wave peak (trailing edge), LRS-feature, time-to-10% of a-wave peak, b-wave amplitude, and time-to-20% of b-wave peak (trailing edge):

$${\text{Temperature}} = - 28.14 \cdot {\text{b}}70{\text{T}} - 2.794 \cdot {\text{LRS}} - 6.069 \cdot {\text{a}}10 - 1.836 \cdot {\text{ba}} + 9.230 \cdot {\text{b}}20{\text{T}} + 66.63\,(^\circ {\text{C}}).$$

The RMS error of this model at cross-validation with training data was 0.36 °C. The generalizability of the constructed model was validated by calculating the temperature estimates given by the model for a new set of photoresponses (test dataset) and comparing those to the actual measured temperatures. The relationship between estimated and measured temperature is illustrated in Fig. 4c. Furthermore, Fig. 4d shows the histogram of error values, giving mean absolute error of 0.28 °C and RMS error of 0.35 °C. When the test set feature values were normalized by only the most recent reference feature value, the mean absolute error and RMS error were equal to 0.34 and 0.44 °C, respectively (Figs. 4c–4d insets).

### Alternative Models with Limited Feature Sets

**Figure 5:**
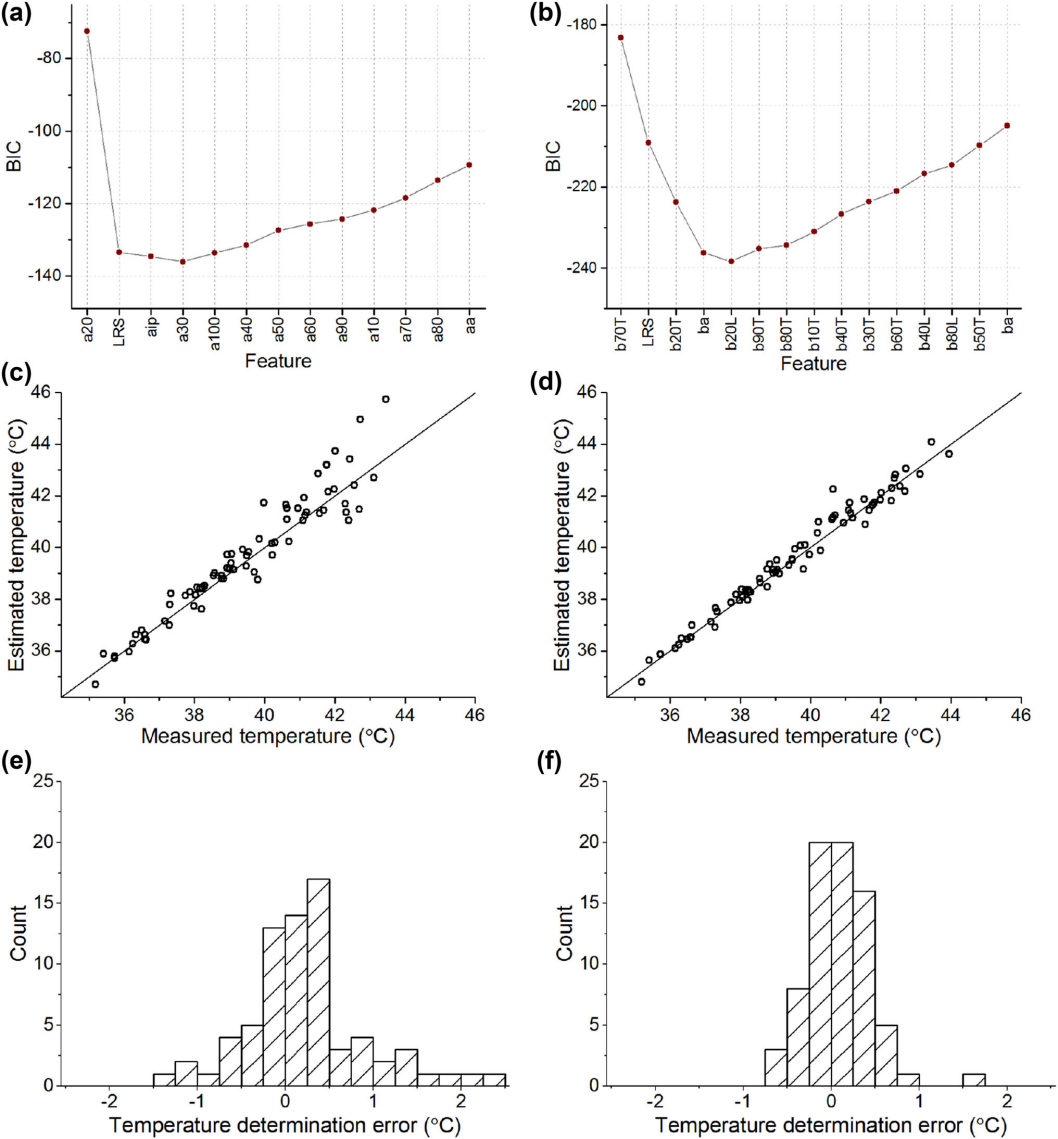
Model development and validation based on limited feature sets. (a) Behavior of BIC value as features are incrementally selected to the final model among OS-features; (b) behavior of BIC value as features are incrementally selected to the final model among dim flash response features; (c) relationship between estimated and measured temperatures, when using the constructed OS-model on new data (test set of 5 retinas, 74 data points). One of the points is outside the range shown, at (43.9, 47.7); (d) relationship between estimated and measured temperatures, when using the constructed dim flash model on new data (test set of 5 retinas, 74 data points, 4 dim flash responses averaged for both stimulus wavelengths). E) Histogram of temperature determination errors (*T*_estimated_ − *T*_measured_) for the OS-model. One of the errors (+3.7 °C) is outside the range shown; (f) Histogram of temperature determination errors for the dim flash response model.

We constructed and tested two alternative temperature determination models. The first one is based on the features whose origin is more purely in the outer segment layer, i.e. bright flash response features and the LRS feature. These features are referred as OS-features and the model is called OS-model. The other model is composed of dim flash response features only and referred as dim flash model. Figures 5a and 5b illustrate the behaviour of BIC values as features were selected to the model among OS-features or dim flash features, respectively. Based on the plot in Fig. 5a, an OS-model with the following four features was selected: time-to-20% of a-wave peak, LRS-feature, time to inflection point of the a-wave, and time-to-30% of a-wave peak:

$${\text{Temperature}} = - 17.81 \cdot {\text{a}}20 - 10.61 \cdot {\text{LRS}} + 12.02 \cdot {\text{aip}} - 18.17 \cdot {\text{a}}30 + 71.59\,(^\circ {\text{C}})$$

Furthermore, based on the plot in Fig. 5b, a model with the following five dim flash features was selected: time-to-70% of b-wave peak (trailing edge), LRS-feature, time-to-20% of b-wave peak (trailing edge), b-wave amplitude, and time-to-20% of b-wave peak (leading edge):

$${\text{Temperature}} = - 31.75 \cdot {\text{b}}70{\text{T}} - 3.593 \cdot {\text{LRS}} + 11.86 \cdot {\text{b}}20{\text{T}} - 2.104 \cdot {\text{ba}} - 3.134 \cdot {\text{b}}20{\text{L}} + 65.75\,(^\circ {\text{C}})$$

The RMS error obtained with the OS-model at cross-validation of the training data was 0.56 °C, and for the dim flash model it was 0.37 °C. The generalization of these models to the test data is illustrated in Figs. 5c–5d, showing the relationship between the estimated and the measured temperatures. For the OS-model, the mean absolute error was 0.57 °C and the RMS error was 0.85 °C, and for the dim flash model, the corresponding errors were 0.27 and 0.37 °C, respectively. The histograms of temperature estimation errors are shown in Figs. 5e–5f.

All the previous test dataset-based validations applied four averaged dim flash responses per wavelength and/or a single bright flash response for each temperature determination. We investigated the effect of reducing the number of averaged dim flash responses to one (i.e. no averaging). As a result, the absolute error and the RMS-error of the dim flash model increased slightly, to 0.30 and 0.40, respectively (data not shown).

## Discussion

Electroretinography is a standard tool in ophthalmological research and diagnostics and it can be recorded both *ex vivo* from an isolated retina and *in vivo* from the surface of the cornea. In this study, we introduce a novel method for retinal and RPE temperature determination based on temperature dependent properties of ERG flash responses: kinetics, absolute sensitivity, and relative sensitivity at long wavelengths. We lay the foundation for the method by determining the temperature dependencies of several features extracted from scotopic *ex vivo* ERG photoresponses, constructing multivariable regression models between the temperature and the feature values, and testing the generalizability of the models on new data.

### Comparison to Previous Data

The temperature dependent properties of photoreceptoral light responses have been studied extensively both by single cell recordings and *ex vivo* ERG (see e.g. Refs. 1,12,21,25), but less is known about the temperature dependencies in the photoresponses of the entire retina. Kong *et al*. and Mizota *et al*. have recorded *in vivo* ERG flash responses from anesthetized mice at normal body temperature and at lower temperatures down to 30 or 18 °C, respectively.^10^,^19^ The data by Kong *et al*. demonstrated an increase in time-to-peak of the b-wave of approximately 3% per 1 °C decrease in body temperature within the temperature range of 35–37 °C, which is close to the temperature dependence observed by us. However, their recordings did not show observable change in a-wave time-to-peak within the temperature range of 35–37 °C. Mizota *et al*. also reported general deceleration of kinetics for lower body temperatures but no numerical values were given. Their recordings demonstrated an increase in b-wave amplitudes of dim flash responses as the body temperature was decreased from 38 to 33 °C, which is in line with our observations.

The temperature dependence of the relative sensitivity at long wavelengths, arising from the physics of visual pigment excitation, can be determined theoretically as described originally in Ref. 14. The minimum energy for photoactivation in mouse rods needed for the calculation is determined in Ref. 15. The theoretical change for the relative sensitivity, defined as *S*_relative_ = *S*(532)/*S*(780), is approximately −3.8% when the temperature increases from 37 to 38 °C. This value closely corresponds to the slope of −3.2% per 1 °C increase obtained for the LRS feature (see “Materials and Methods” for definition) in our recordings. To our knowledge, no previous data exists on the temperature dependence of long wavelength relative sensitivity by ERG recordings with intact rodents or other animals.

### Features and Model Development

ERG flash responses are generated as a superposition of several components that originate in different sources in the retina: photoreceptors, bipolar cells, Müller cells, and the inner retina, all of them having individual activation and deactivation kinetics and temperature dependencies. The contribution of each component is dependent on flash strength and the temporal location along the ERG response trace. Therefore, we extracted several time-to-*X*% features from both dim and bright flash responses. These features are quite redundant, but they provide a comprehensive representation of the kinetics of the whole response. Feature selection was applied to choose the kinetics features that, based on the training data, would give the most accurate temperature determination. Overall, the feature selection among similar features is prone to randomness and replacing a feature with a resembling one is likely to have little effect on the accuracy of the model. On the other hand, it is beneficial for the model to consist of distinct features that are less redundant and provide supplementary information. This applies for example to the LRS-feature, which is based on a phenomenon independent of all other features.

Considering the application of the method for heating treatments, the initial phase of the a-wave (before the b-wave starts to overlap) is likely to represent the temperature of the RPE closely because it originates in the outer segments of photoreceptors partially embedded in RPE. With this in mind, the bright flash response features were determined from the leading edge of the a-wave. Moreover, the LRS-feature is expected to give accurate information on RPE temperature despite its determination being based on the b-wave, because the phenomenon itself takes place in the OS, and other temperature-related changes in the response amplitude are canceled by normalization with the 532 nm flash response. Consequently, we developed an OS-model which consists of the features mentioned above and should represent the temperature of the RPE closely. On the other hand, the benefit of using dim flash responses in temperature determination is that they can be recorded with high repetition rate and used to produce both high-accuracy kinetics features and the LRS-feature. Thus, we also constructed and tested a model consisting of dim flash response features only. If the heating treatment induces strong retinal temperature gradients, the accuracy for estimating RPE temperature with this model may decrease due to b-wave being affected by the temperature of the bipolar cell layer and even that of the inner retina through the slow PIII component.

Absolute response kinetics and sensitivity vary among individuals. Therefore, we operated with relative feature values that were normalized by the corresponding values at 37.0 °C of each individual. By employing this kind of calibration, the method determines the change of retinal temperature compared to the initial value. When applying the temperature determination method for heating treatment, this individual calibration can easily be performed before the heating. Additionally, if the temperature estimate is calculated as a moving average, there exists a tradeoff between the temporal resolution and the accuracy of the determination. According to our results, reducing the number of averaged dim flash responses from four to one had only a slight effect on the accuracy of the dim flash model. In the case of *in vivo* mouse ERG, using one dim flash response for each temperature estimate would enable a temporal resolution even higher than 1 s. A useful feature has both monotonic and steep temperature dependence and high precision (i.e. low variance at each temperature). The extracted features generally showed worse precision and increased variance towards higher temperatures. As a result, the temperature estimation errors of all models tend to grow towards higher temperatures.

### Applicability of the Proposed Method for *In Vivo* Use

When considering the applicability of the method for intact mice, at least three questions must be addressed: (1) Do the features determined from *in vivo* ERG responses have monotonic and steep enough temperature dependencies across the targeted temperature range? (2) Does the quality of *in vivo* recording (signal-to-noise ratio) allow retinal temperature to be determined precisely enough for heating treatments? (3) How closely does the ERG response, originating in the distal retina, reflect the temperature of the RPE during a heating treatment of an intact eye?

Previous data with mouse *in vivo* ERG together with theoretical considerations indicate that qualitatively similar temperature dependencies exist in *in vivo* ERG responses compared to the *ex vivo* recordings obtained in this study, enabling a similar temperature determination method to be applied with intact mice. However, further experimental work is required to define the precise temperature dependencies *in vivo* for the calibration of the temperature determination model.

While *ex vivo* ERG responses have a high signal-to-noise ratio, similar responses recorded *in vivo* are more prone to biological noise and movement artefacts affecting the accuracy of retinal temperature determination. These can be minimized by signal processing and careful planning of the mechanical setup. On the other hand, ERG photoresponses (especially the b-wave) recorded *in vivo* stay more stable over long time periods compared to *ex vivo* ERG, which improves the accuracy of temperature determination.

The similarity of the temperatures of RPE and distal retina during a heating treatment can be assessed by modeling the temperature distribution in the retina. According to the existing models and theoretical considerations, the axial temperature distribution across the retina, RPE, and choroid becomes more even as the heating time increases, favoring the application of the proposed method for lengthy heating treatments.^17^,^26^ Temperature distribution has also been addressed by direct temperature measurements. Ibarra *et al*. monitored the retinal temperatures of anesthesized pigmented rabbits by ultrafine thermocouple during 60-s heating sessions where temperature was raised 2–5 °C by transpupillary heating with 810 nm laser light.^9^ They did not observe statistical difference between the temperatures recorded from the inner limiting membrane of the retina and from the subretinal space between outer segment layer and the RPE suggesting that no strong radial temperature gradients were present in their heating setup.

As a summary, the temperature determination method demonstrated in this paper would be beneficial for the RPE heating treatment approaches that use lengthy exposures and moderate temperature elevations. The RMS error level for retinal temperature determination obtained with the *ex vivo* ERG recording was 0.35–0.40 °C, which clearly meets the needs of heating treatment approaches. Temperature dependencies in the ERG signal kinetics and amplitudes as well as in the long-wavelength relative sensitivity have been reported also for humans,^3^,^8^ suggesting that a similar approach could be applied in clinical heating treatments. However, instead of rod responses applied here with the rod dominant mouse retina, it might be appropriate to use the temperature dependent features of cone responses of human ERG. In cones the photoresponse recovery is faster allowing higher light stimulation rate, which improves the temporal and temperature resolution of retinal temperature determination.

## Abbreviations

ERG: Electroretinography
RPE: Retinal pigment epithelium
R*: Isomerized rhodopsin
RMS(D): Root mean square (deviation)
OS: Outer segment of photoreceptor
BIC: Bayesian information criterion
